# Molecular Dynamics Investigation of Oil Wetting on Synthetic Polymer Substrates

**DOI:** 10.1021/acs.macromol.6c00947

**Published:** 2026-07-10

**Authors:** Hang Zhang, Sahana V. Sundar, Shawn M. Maguire, Rodney D. Priestley, Emily C. Davidson, Michael A. Webb

**Affiliations:** † Department of Chemistry, 6740 Princeton University, Princeton, New Jersey 08540, United States; ‡ Department of Chemical and Biological Engineering, 6740 Princeton University, Princeton, New Jersey 08540, United States; ¶ Princeton Materials Institute, 6740 Princeton University, Princeton, New Jersey 08540, United States

## Abstract

Understanding the interaction of polymer surfaces with nonpolar, low surface tension liquids, or whether a substrate is oleophobic versus oleophilic, is critical for applications ranging from antifingerprint coatings to oil–water separation membranes and oil spill remediation. Despite its technological importance, the molecular mechanisms underpinning polymer–oil wetting are not well characterized. Here, we employ molecular dynamics simulations to investigate the behavior of n-hexadecane in contact with chemically distinct polymer surfaces, spanning eight constitutional unit chemistries as well as amorphous and crystalline morphologies. This permits a critical examination of both thermodynamic and dynamic descriptors of oleophobicity, including oil contact angle, dewetting free energy, interfacial diffusivity, and a proposed “ghost probe energy,” which does not require explicit simulation of oil–polymer interactions. We find that the oil contact angle does not reliably distinguish oleophobic behavior across polymer chemistries, whereas other metrics provide clearer and more consistent differentiation. Analysis of the results reveals that polymer–oil wetting behavior is primarily governed by interfacial van der Waals interactions and modulated by surface flexibility and morphology. Collectively, this work establishes a computational framework for characterizing oil wetting and provides additional insight into what molecular-level factors dictate trends in oleophobicity.

## Graphical abstract

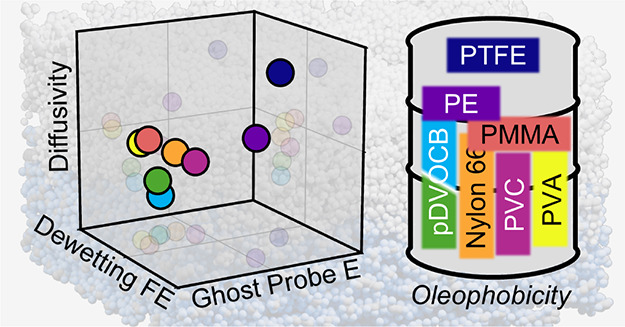

## 1 Introduction

Oil wetting behavior is commonly described in terms of oleophobicity, which refers to the tendency to repel liquids with low surface tension, such as mineral oil, gasoline, and sebum.^1–6^ Similarly to hydrophobicity,^7–9^ which refers to interaction with water, oleophobicity plays a pivotal role in modern surface technologies and applications. For example, high oleophobicity is crucial for antifingerprint coatings of modern smart devices;^10–12^ low oleophobicity (alternatively, high oleophilicity) is central to oil separation for recovery and environmental protection;^13–16^ and detailed control of oleophobicity is important for self-transportation and self-assembly of materials.^17–20^ In recent decades, more complex oil-wetting phenomena on polymer surfaces, including dynamic wetting^21–25^ and wetting on liquid-like surfaces (LLSs),^26–28^ have also been extensively studied. The breadth of these applications and investigations underscores the importance of understanding the factors that govern oleophobic behavior at surfaces.

One key factor influencing oil wetting is surface chemistry. Per- and polyfluoroalkyl substances (PFASs) exemplify this relationship, exhibiting exceptionally strong oleophobicity^29^ that, combined with high chemical stability and low biological toxicity, has led to their widespread use in chemical, electronic, construction, and automotive industries.^30–32^ However, PFASs have raised serious concerns about negative environmental influence,^33–36^ including water pollution,^37–39^ biosphere disruption,^40–42^ and human health effects.^43–45^ These environmental concerns motivate the development of fluorine-free alternatives^46–50^ but with similar functional performance. More broadly, systematic characterization of oil wetting across chemically diverse polymer surfaces would elucidate the molecular origins of oil-repellent phenomena and provide a foundation for rational materials design.

Surface patterning provides another means to modulate oil wetting.^1,4,5^ This is manifest through distinct wetting regimes, most notably the Wenzel and Cassie–Baxter states.^51,52^ Hierarchically structured surfaces are found in biological systems, such as lotus leaves^53–55^ and springtail skin,^56–58^ and have inspired artificial surface patterns, including micropillars,^59–62^ nanoneedles,^17,63–65^ and nanoflakes,^66–69^ to achieve superoleophobic behavior. Indeed, patterning strategies are viewed as essential for achieving strong oleophobicity because the low surface tension of oils seemingly limits achievable contact angles on flat surfaces.^5,70,71^ While the basis for this limitation is understood from Young’s equation, the molecular-level factors that govern oleophobicity across diverse surface chemistries remain less well characterized. Additionally, fabricating nanoscale architectures is often complex, structures may suffer from limited mechanical robustness,^72–74^ and effectiveness still strongly depends on the intrinsic oleophobicity of the underlying flat surface.^1,4,5^ Consequently, quantitative characterization of oil wetting on controlled flat surfaces enables intrinsic chemical contributions to be isolated from geometric effects, providing fundamental insight into the molecular interactions that govern oil-repellent behavior.

Molecular dynamics (MD) simulations are well suited to provide microscopic insights into surface interactions that influence oleophobicity.^49,75–81^ For example, Meng et al.^77^ investigated the wetting process of heavy oil mixtures on rough silica surfaces, and Zhao et al.^80^ examined the wetting behavior of *n*-octane on smooth and patterned PTFE surfaces. However, these prior studies employ rigid surface models, which can introduce artifacts for soft polymer materials by not capturing the interplay of both chemical and mechanical factors at the interface.^82^ Ultimately, systematic MD investigations of oleophobicity across chemically diverse, flexible polymer surfaces remain scarce.

In this work, we investigate the oil wetting of eight chemically distinct polymers using molecular dynamics simulations and supporting experimental characterization. In particular, we examine hexadecane as a model oil (Figure 1A,B) in combination with six commodity polymers–poly­(tetrafluoroethene) (PTFE), polyethylene (PE), poly­(vinyl chloride) (PVC), poly­(methyl methacrylate) (PMMA), Nylon-66 (N66), and poly­(vinyl alcohol) (PVA)–as well as two recently developed chemically recyclable polymers–pDVOCB­(5,40) and pDVOCB­(6,46) (Figure 1C); these latter polymers are derived from (1,*n′*-divinyl)­oligocyclobutanes that are then chain-extended using acyclic diene metathesis (ADMET) polymerization^83^ to yield a family pDVOCB­(*n*,*m*), where *n* refers to the number of cyclobutane units in an oligomer and *m* is the number of enchained DVOCB­(*n*) units. For notational convenience, we refer to pDVOCB­(5,40) and pDVOCB­(6,46) as pD5 and pD6 throughout the remainder of the text. Oil wetting is characterized using multiple oleophobicity metrics, including contact angle, dewetting free energy, a newly defined ghost probe energy, and diffusivity. Across all metrics, PTFE exhibits the highest oleophobicity, consistent with its well-known oil-repellent properties. Analysis reveals that surface vdW interaction strength plays a dominant role in governing oleophobicity and that this quantity can be probed as an efficient and predictive metric. Additionally, oil diffusion behavior differs substantially between amorphous and crystalline surfaces, with ordered channel structures emerging on crystalline substrates that may influence directional liquid transport. Overall, these findings provide molecular-level insights into the factors governing oil wetting on polymer substrates and suggest design principles for fluorine-free oleophobic materials.

**Figure 1:**
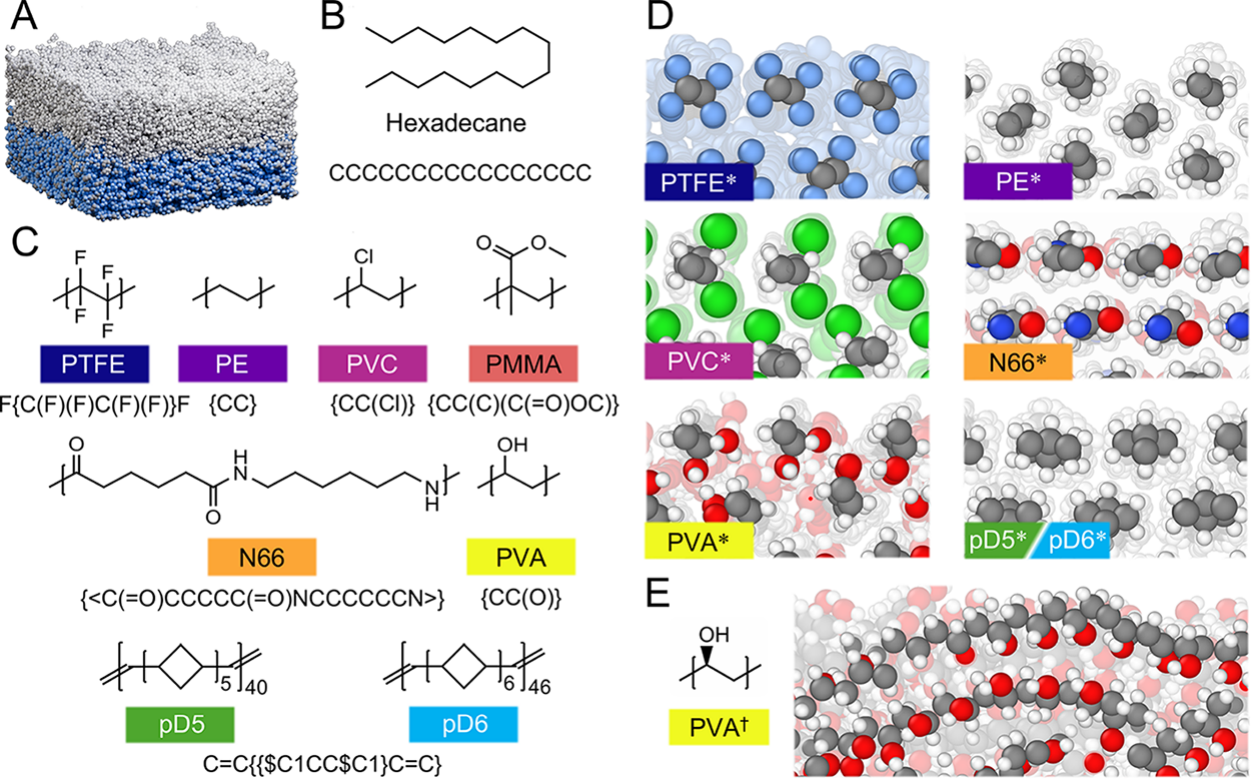
Overview of systems. (A) Characteristic system set up, as demonstrated through molecular rendering of PTFE-oil system. (B) Chemical structure of hexadecane, which is used as the model oil. (C) Chemical structure, reference names, and BigSMILES strings for polymers. The reference names are shown inside a colored block. The same colors are used in subsequent figures. (D) Additional crystalline surfaces of seven polymers (indicated by *). Crystalline surface of PMMA is not studied for its predominantly amorphous character and lack of a well-defined crystalline unit cell. (E) Isotactic PVA surfaces (indicated by PVA^†^). Molecular images are visualized using Blender^84^ and OVITO.^85^ The elements are colored such that carbon is gray, fluorine is blue, chlorine is green, nitrogen is dark blue, oxygen is red, and hydrogen is white. One snapshot of pDVOCB­(6,46) crystal is shown to represent the side view of pDVOCB­(5,40) and pDVOCB­(6,46) crystals.

## 2 Methods

### 2.1 Overview of Systems and Methods

As shown in Figure 1, the oil-surface interactions between the model oil, hexadecane, and 16 distinct polymer surfaces are simulated: nine amorphous polymers (PTFE, PE, PVC, PMMA, N66, PVA, pD5, pD6, and PVA^†^) and seven crystalline polymers (PTFE*, PE*, PVC*, N66*, PVA*, pD5*, and pD6*) (Figure 1D). For polymers with tacticity, PVC, PMMA, PVA, and PVA* are atactic; PVA^†^ is isotactic (Figure 1E); and PVC* is syndiotactic. These chemically distinct polymers are either technologically important or scientifically novel. In addition, nine other surfaces are studied to assess robustness of certain analyses. This includes a “frozen” amorphous surface of PE (PE°), in which the surface atoms are fixed in place; this informs on the role of mechanical deformation. The remaining eight surfaces are based on pDVOCB (four amorphous and four crystalline). These surfaces are denoted as pD5_g_, pD5_s_, pD6_g_, and pD6_s_, for the amorphous systems, and pD5_g_^*^, pD5_s_^*^, pD6_g_^*^, and pD6_s_^*^, for the crystalline systems. The subscripts indicate the use of alternative parameter-sets for modeling pDVOCB-hexadecane interactions (see also Section 2.3); the results for these may be interpreted as a type of sensitivity analysis relevant to uncertainties in the modeling workflows. For amorphous polymer systems, three independent polymer slabs are prepared to assess statistical uncertainty. One polymer slab is prepared for crystalline polymers based on experimentally determined crystalline structures.^86–91^ Overall, 25 distinct surfaces of eight polymers are investigated in the simulations.

Across the 25 surfaces, we consider idealized morphologies of both amorphous and crystalline surfaces. In experimental settings, many polymers are semicrystalline, with surfaces composed of both amorphous and crystalline regions. Here, we simulate purely amorphous and crystalline surfaces as two limiting cases of local polymer surface structure. This distinction matters because morphology can alter surface chemical accessibility, rigidity, and nanoscale roughness. Previously, we found that amorphous-to-crystalline changes can substantially affect water wetting based on modulation of interfacial interactions that largely depended on hydrogen-bonding.^82^ It remains unclear whether such morphology-dependent effects are equally significant for oil wetting where hydrogen bonding is expected to be less significant, motivating our consideration of both amorphous and crystalline surfaces.

For each polymer surface, four oleophobicity metrics are calculated: the simulated nanoscale oil contact angle, the oil dewetting free energy βΔ*f*_σ_^o^, the ghost probe energy β*E*_gp_, and the interfacial diffusion coefficient; the ghost probe energy is a metric unique to this work and described in Section 2.4.3. For contact angle simulations, an oil droplet is deposited atop a polymer slab. Otherwise, the system consists of a polymer slab and an oil slab, as shown in Figure 1A. Additionally, we performed experimental oil contact angle measurements for real polymer samples to compare with our simulation results.

### 2.2 General Simulation Details

All MD simulations are run using version 3 Mar 2020 of LAMMPS simulation package.^92^ Oil (hexadecane) and PE are described using the LOPLS-AA force field.^93,94^ Other commodity polymers are described using the OPLS-AA force field.^95^ The DVOCB-based polymers are described using the TAFFI force field,^96^ with modifications described in prior work.^97^ To handle the mixing of force fields required for oil-pDVOCB interactions, three different mixing rules were tested (Section 2.3). Real-space nonbonded interactions are truncated at 10 Å for commodity polymers, while a larger cutoff of 14 Å is employed for pDVOCB polymers to accommodate the longer-range interactions associated with the TAFFI force field parametrization. To account for the slab geometry, simulation cells are periodic in **x̂** and **ŷ** but nonperiodic in **ẑ**. Long-range electrostatics are handled using the particle–particle particle-mesh Ewald^98^ summation method with a 10^–5^ convergence accuracy and modification for the nonperiodic z-dimension.^99^ Unless otherwise stated, the temperature is controlled at 300 K using a Nosé–Hoover thermostat^100^ with a damping constant of 100 fs.

Surface preparation follows procedures described in previous studies.^82,101^ The crystalline surfaces are constructed from the (100) surface of phase IV crystalline PTFE,^86^ the (010) surface of crystalline PE,^87^ the (100) surface of crystalline PVC,^88^ the (010) surface of crystalline N66,^89^ the (100) surface of crystalline PVA,^91^ and the (100) surface of the monoclinic unit cell of pDVOCB polymers. We note that all crystalline surfaces considered in this study adopt an orientation in which polymer chains lie parallel to the surface. In real materials, crystalline domains can exhibit a range of orientations, which may influence the oleophobicity of the surface. To form “droplets” of oil, 512 hexadecane molecules are distributed on surfaces of approximately 260 × 260 Å. For simulations in pure slab geometries, 1200 hexadecane molecules are deposited on surfaces of approximately 130 × 130 Å. For simulations with droplets, simulations of up to 8 ns are performed in the canonical (NVT) ensemble to calculate contact angles. For simulations with slabs of polymer/oil phases, the system is first equilibrated for 10 ns in the NVT ensemble, then the resulting configurations are used to calculate oil dewetting free energies using the INDUS extension^102^ in the PLUMED library.^103^ Additional simulations of 3 ns are performed in the NVT ensemble to calculate diffusion coefficients and density distributions of hexadecane molecules. Finally, to calculate ghost probe energy, simulations are run with 5 ns of equilibration and 5 ns of production, with only polymer surfaces (approximately 130 × 130 Å) are performed in the NVT ensemble.

### 2.3 Mixing Rules of Oil-pDVOCB Interactions

To simulate the oil–pDVOCB systems, we evaluated three distinct Lennard–Jones (LJ) mixing rules to bridge the LOPLS-AA force field of the oil phase and the TAFFI force field of pDVOCB polymers.

The Waldman-Hagler rule, which is the default mixing rule in the TAFFI force field,^96^ is defined as

$$\epsilon_{ij}=\frac{2\sqrt{\epsilon_{i}\epsilon_{j}}\sigma_{i}^{3}\sigma_{j}^{3}}{\sigma_{i}^{6}+\sigma_{j}^{6}}$$ 1

$$\sigma_{ij}={\left(\frac{1}{2}\left(\sigma_{i}^{6}+\sigma_{j}^{6}\right)\right)}^{1/6}$$ 2

The geometric rule, which is the default mixing rule in the OPLS force field family,^93–95^ is defined as

$$\epsilon_{ij}=\sqrt{\epsilon_{i}\epsilon_{j}}$$ 3

$$\sigma_{ij}=\sqrt{\sigma_{i}\sigma_{j}}$$ 4

In addition, we introduced a third scheme, referred to as the scaled Waldman–Hagler rule, in which the ϵ_*ij*_ values from the Waldman–Hagler rule are multiplied by an element-specific scaling factor. These scaling factors were calibrated to reproduce the DFT pair energy of DVOCB(3)–hexadecane dimers at the same level of theory used in the TAFFI parametrization. This modification strengthens the oil–pDVOCB interactions relative to the original Waldman–Hagler rule. More details are in Section S1 of the Supporting Information.

All oleophobicity metrics were computed using each of the three mixing rules. Considering the underlying chemistry of the pDVOCB polymers, the Waldman–Hagler rule yields the most physically reasonable trends. The scaled Waldman–Hagler rule gives overly weak oleophobicity relative to other polymers, while the geometric rule leads to less consistent behavior in oil diffusivity on crystalline surfaces. Accordingly, surfaces constructed with the Waldman–Hagler rule are treated as the reference polymer surfaces (pD5, pD6, pD5*, and pD6*). Surfaces generated using the geometric rule (pD5_g_, pD6_g_, pD5_g_^*^, and pD6_g_^*^) and the scaled Waldman–Hagler rule (pD5_s_, pD6_s_, pD5_s_^*^, and pD6_s_^*^) are treated as model surfaces for additional comparison.

### 2.4 Analysis of Oleophobicity

#### 2.4.1 Contact Angles

##### Simulation

For all polymer surfaces except PTFE, hexadecane completely wets the surface, which obfuscates extraction of any contact angle. For PTFE surfaces, a nonzero oil contact angle is observed, and we use a method based on arc fitting of the average density in r-z plane to calculate the oil contact angle. Details of the calculation can be found in our previous work.^82^ The last 2 ns simulations are used to calculate the average density distribution.

##### Experiment

Contact angle measurements were taken using a Krüss DSA30E Drop Shape Analyzer. A computer-controlled glass syringe was used to deposit a 5 μL drop of hexadecane on the sample surface. After adding a manual baseline, each captured image of each droplet was automatically analyzed by the Krüss Advance software to calculate the contact angle. For each analyzed polymer, at least five separate droplets were measured at unique surface locations. The measured contact angles were then corrected with the Wenzel equation, using the average rugosity factor measured via AFM, in order to calculate the Young’s contact angle. We note that the droplets of oil were larger than the roughness scale by at least two to three orders of magnitude, validating the application of the Wenzel equation. Details about the samples and experiments are in Section S2 of the Supporting Information.

#### 2.4.2 Oil Dewetting Free Energy

The indirect umbrella sampling (INDUS) method is used to calculate the dewetting free energy of hexadecane from polymer surfaces. The approach follows analogously to that used for calculating dehydration free energies.^82,104^ Briefly, a series of bias potentials ψ is applied to control a collective variable *Ñ*_*v*_^o^, which here corresponds to the number of hexadecane carbon atoms within a controlled volume. The volume *v* is defined as a cuboid of dimensions 50 × 50 Å × (⟨*z*_0_⟩ + 8) Å, where ⟨*z*_0_⟩ is the vertical position of the hexadecane interface, defined as the height at which the hexadecane density reaches half its bulk value. The free energy required to completely dewet the volume is calculated by

$$F_{v}\left({\left\langle {Ñ}_{v}^{o}\right\rangle }_{\psi }\right)=-\frac{1}{\beta }\ln\;P_{v}^{\psi }\left({\left\langle {Ñ}_{v}^{o}\right\rangle }_{\psi }\right)-\psi {\left\langle {Ñ}_{v}^{o}\right\rangle }_{\psi }+\int_{0}^{\psi }{\left\langle {Ñ}_{v}^{o}\right\rangle }_{\psi \prime }d\psi^{\prime }$$ 5

Here, β = (*k*_B_ *T*)^−1^, ⟨*Ñ*_*v*_^o^⟩_ψ_ is the average smoothed number of hexadecane carbon atoms in *v*, and *P*_*v*_^ψ^(⟨*Ñ*_*v*_^o^⟩_ψ_) is the probability of observing ⟨*Ñ*_*v*_^o^⟩_ψ_ in an ensemble subjected to a biasing potential ψ.

Because flexible polymer surfaces can intermix with hexadecane to varying extents, the unbiased hexadecane content ⟨*Ñ*_*v*_^o^⟩_0_ differs across surfaces. To account for this variation, the dewetting free energy *F*_*v*_(0) is normalized by ⟨*Ñ*_*v*_^o^⟩_0_ to yield our oleophobicity metric βΔ*f*_σ_^o^:

$$\beta \Delta f_{\sigma }^{o}=\beta F_{v}\left(0\right)/{\left\langle {Ñ}_{v}^{o}\right\rangle }_{0}$$ 6

For all surfaces, an array of biasing potentials ψ = [0.0, 0.4, 0.8, 1.0, 1.2, 1.4, 1.6, 1.8, 2.0] kJ/mol is employed. For pDVOCB model surfaces with the scaled Waldman-Hagler rule, additional simulations with ψ = [2.2, 2.4, 2.6] kJ/mol are performed to overcome the strong oil-surface interaction and completely dewet the surface. Simulations of 2.5 ns are run iteratively until *Ñ*_*v*_^o^ is stable for the final 2 ns; the shortest biasing simulations are 5 ns and the longest (at large ψ) are 17.5 ns.

#### 2.4.3 Ghost Probe Energy

We introduce a new metric that we refer to as the ghost probe energy, β*E*_gp_, and examine its correlation with more intensive characterizations of oleophobicity. In effect, the ghost probe energy quantifies nonbonded dispersion interactions with a given polymer substrate; its evaluation is facilitated by placing a grid of “ghost” particles above the surface and computing characteristic interaction energies. Importantly, ghost particles interact with polymer atoms via a conventional nonbonded Lennard-Jones (LJ) potential; ghost particles do not interact with other ghost particles. Furthermore, because this calculation does not require explicit hexadecane molecules in the simulation, it provides a computationally efficient approach for screening polymer oleophobicity that requires only polymer surface configurations.

To obtain a characteristic ghost probe energy, NVT simulations of 5 ns are first performed with only the flexible polymer surface present to sample system configurations. From these trajectories, ⟨*z*_p_⟩, the height at which the polymer density reaches half its bulk value, is calculated to define the polymer interface position. A two-dimensional grid of ghost particles is then placed at varying heights above the surface. For amorphous and crystalline systems, grid spacings of 1 and 0.5 Å, respectively, are sufficient to obtain converged results. The grid height *z* is varied from ⟨*z*_p_⟩– 20 Å to ⟨*z*_p_⟩ + 12.5 Å in increments of 0.5 Å.

By rerunning the surface trajectory with ghost particles at height *z*, the LJ energy *E*_*i*,*t*_^α^(*z*) between ghost particle *i* and the surface at time *t* is calculated using the OPLS-AA parameters for atom type α. From these values, the Boltzmann-weighted average energy for atom type α at height *z* is computed as

$$E^{\alpha }\left(z\right)=\frac{\underset{i,t}{\sum }E_{i,t}^{\alpha }\left(z\right)\exp\left[-\beta E_{i,t}^{\alpha }\left(z\right)\right]}{\underset{i,t}{\sum }\exp\left[-\beta E_{i,t}^{\alpha }\left(z\right)\right]}$$ 7

Here, α corresponds to some representative atom types; we use the atom types C81L (carbon) or H85LCH2 (hydrogen) in the LOPLS-AA force field. With these atom types, we then evaluate a *z*-dependent quantity

$$E^{{\mathrm{CH}}_{2}}\left(z\right)=E^{C}\left(z\right)+2E^{H}\left(z\right)$$ 8

which is representative of interaction with a methylene group characteristic of hexadecane. Examples are shown in Section S4 of the Supporting Information.

Finally, the ghost probe energy β*E*_gp_ is calculated with another Boltzmann-weighted average as

$$\beta E_{\mathrm{gp}}=\frac{\int_{z_{0}}^{z_{1}}\beta E^{{\mathrm{CH}}_{2}}\left(z\right)\exp\left[-\beta E^{{\mathrm{CH}}_{2}}\left(z\right)\right]dz}{\int_{z_{0}}^{z_{1}}\exp\left[-\beta E^{{\mathrm{CH}}_{2}}\left(z\right)\right]dz}$$ 9

Here, z_0_ and z_1_ are defined as ⟨*z*_p_⟩ and ⟨*z*_p_⟩ + 10 Å. The integration is performed numerically through a spline interpolation of *E* ^CH_2_^(*z*). Overall, this ghost probe energy provides a general metric to describe the LJ interaction strength above both amorphous and crystalline surfaces.

#### 2.4.4 Oil Diffusivity

We calculate the self-diffusion coefficient of oil molecules on the surface in the **x̂**, **ŷ**, and **ẑ** directions through mean squared displacement (MSD) of the center of mass of oil molecules. The MSD is calculated based on oil molecules that have a height lower than (⟨*z*_0_⟩ + 8 Å) at the beginning. A linear function is fit to the MSD between 0.1 and 1.0 ns to calculate the self-diffusion coefficient. This time window is chosen to exclude subdiffusive dynamics at short time scales while minimizing events in which molecules leave the interfacial region. We also calculate the self-diffusion coefficient in the bulk oil of 1200 molecules, which gives a result of 4.65 × 10^–6^ cm^2^/s, in reasonable agreement with the experimental value, 4.1 × 10^–6^ cm^2^/s.^105^

## 3 Results and Discussion

### 3.1 Contact Angle as a Metric for Oleophobicity

The contact angle of an oil-based liquid with a surface might be generally expected to function as a straightforward metric of oleophobicity.^4,5,107^ In a previous study on polymer hydrophobicity, we found good correspondence between contact angles measured from simulations of nanoscale water droplets and those obtained from macroscopic experimental measurements, with trends resonant with expectations of the functional-group chemistry.^82^ This motivated the present investigation to characterize hexadecane contact angles across the various polymer surfaces and assess alignment between simulation and experiment (Figure 2). We note that prior studies have shown that soft polymeric substrates can exhibit behavior beyond partial or complete wetting, including liquid uptake, mixing, or even dissolution when polymer–liquid affinity is sufficiently strong.^108,109^ Experimentally, we did not observe clear evidence of polymer dissolution, and in simulations, the polymer substrates maintained their slab geometries over the simulated time scales.

**Figure 2:**
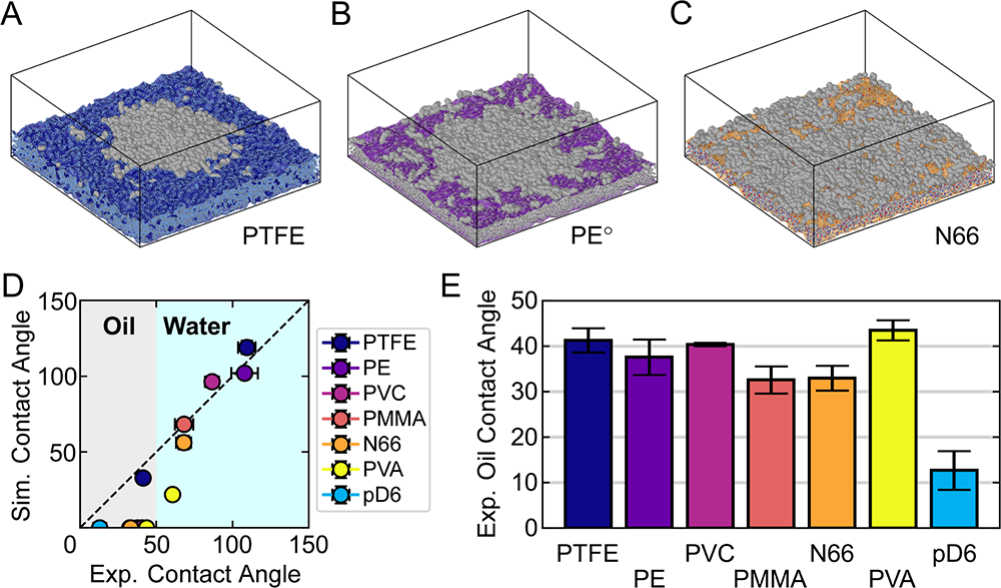
Comparison of oil wetting on polymer surfaces. Simulation snapshots of wetting on (A) PTFE, (B) PE°, and (C) N66. To help distinguish the surface and oil, a visual mesh is created based on surface atoms, and carbon atoms of hexadecane are enlarged. (D) Comparison between simulated and experimentally measured contact angles for hexadecane (gray region) and water (cyan region). All values for oil are from this work, simulated contact angles with water are from ref 82, and experimental contact angles with water are from PolyInfo.^106^ There is no experimental data for pD5 because there was inadequate material. For simulation results, error bars reflect statistical uncertainties reported as the standard error of the mean from three independent simulations. For experimental results, error bars of experimental oil and water results reflect statistical uncertainties reported as the standard deviation from independent experiment measurements and PolyInfo data set, respectively. (E) Experimentally measured contact angles with hexadecane. The error bars reflect statistical uncertainties reported as the standard deviation from independent experiment measurements. The molecular images are visualized using OVITO.^85^ The blue, purple, and gold surfaces in the molecular renderings are constructed from the identified surface atoms, which indicates the interface positions. The elements are colored such that carbon is gray, fluorine is blue, nitrogen is dark blue, oxygen is red, and hydrogen is white.

In our simulations, contact angle is not a sensitive metric for surface oleophobicity across the considered polymers. This is because all polymers except PTFE are completely wet by hexadecane (Figure 2C), precluding extraction of a contact angle. We note that contact angles are calculated from equilibrated oil droplets on polymer surfaces and are interpreted as static sessile-droplet contact angles for systems that retain nonzero values. Systems with zero contact angle correspond to complete wetting. Only three systems yield a stable, nonzero contact angle: 33.0° ± 1.3° for the amorphous PTFE surfaces, somewhat lower 23.2° for the crystalline PTFE surface, and 17.9° ± 0.7° for the frozen amorphous PE model surfaces. Even so, these values are relatively low compared to contact angles typically associated with strongly oleophobic behavior. However, these results are qualitatively consistent with thermodynamic expectations. In particular, the low surface tension of hexadecane leads to complete wetting on most surfaces, with only the lowest surface energy material exhibiting measurable oleophobicity. However, this consistency does not translate into quantitative utility, since there is no variability among the surfaces that are sufficiently oleophilic to be completely wet. By contrast, contact angles could more sensitively rank-order hydrophobicity across a similar set of polymers (Figure 2D).

Experimental contact angle measurements reveal a similar challenge (Figure 2D,E). Although nonzero contact angles are observed for all polymers at the macroscopic scale, the values cluster within a narrow range of approximately 30° to 45°, making it difficult to discern meaningful differences among surfaces. As previously noted, PTFE is generally characterized as oleophobic, and indeed it exhibits among the highest contact angles with hexadecane. Previously reported values for hexadecane on PTFE are typically in the range of 30°–50°,^110–113^ which is consistent with our results. However, the values determined here are statistically similar to several other polymers, including the hydrophilic PVA. Across the other polymers (excluding the pDVOCB-based polymer, pD6), reported contact angles^112–117^ are generally lower than 30° or correspond to complete wetting, which agrees with our simulation results but differs from our experimental measurements. However, detailed descriptions of sample preparation, surface morphology, and measurement conditions are lacking across the literature, making it difficult to identify the origin of these differences. Within the context of our own measurements, pD6 exhibits a lower contact angle than the other polymers, including PE, despite both being purely hydrocarbon-based. This could arise from oxidation or cross-linking of pD6 during melt-processing, which would not be captured by the simulations; however, this was not confirmed, and the origin of this result remains unclear. Ultimately, the distinction between surfaces is difficult to rationalize from contact angle data alone, especially when contextualized with its limited discriminatory power. This aspect becomes more apparent when comparing hexadecane contact angles over a range that includes water contact angles (Figure 2D).

Considering the relationship with simulated contact angles in Figure 2D, we observe some systematic propensity for simulated values to underestimate experimental values for both liquids (water and hexadecane) in the limit of lower contact angles. This trend is more consequential for hexadecane, where the absolute values are already small, but is also evident for PVA-water systems. Although we acknowledge the possibility of some force-field inaccuracies or even difficulties in experimental measurement, some underestimation of contact angles in simulations is also expected from the modified Young’s equation,^118^

$$\cos\;\theta =\frac{\gamma_{\mathrm{SG}}-\gamma_{\mathrm{SL}}}{\gamma_{\mathrm{LG}}}-\frac{\sigma }{R\gamma_{\mathrm{LG}}}$$ 10

where γ_SG_, γ_SL_, and γ_LG_ denote the surface–gas, surface–liquid, and liquid–gas surface tensions, respectively, σ is the line tension at the three-phase contact line, and *R* is the contact radius.

For nanoscale droplets, the line-tension term could become significant. To test whether this line-tension term substantially affects our contact-angle simulations, we performed additional cylindrical droplet geometry simulations for amorphous and crystalline PTFE and PVC systems (Supporting Information, Figure S6). This geometry has been shown in prior works to reduce finite-size and line-tension effects.^119,120^ In these simulations, PTFE systems retain nonzero contact angles that differ by less than 1° from those obtained using the spherical-droplet geometry, whereas PVC again exhibits complete wetting. Thus, line-tension effects appear to have only a minor influence on the contact-angle results for the systems considered here. Taken together, these results demonstrate that contact angle is neither a reliable nor sensitive metric for differentiating oleophobicity among flat polymer surfaces, motivating the investigation of alternative characterization approaches.

### 3.2 Thermodynamic Characterization of Oleophobicity

Previously,^82^ we determined that dehydration free energy could be used as a thermodynamically grounded metric that correlated well with experimental contact angles and successfully distinguished among polymer surfaces. Motivated by this success, we constructed an analogous quantity for oleophobicity, the oil dewetting free energy βΔ*f*_σ_^o^, which quantifies the free energy cost of removing hexadecane from the polymer interface. We hypothesized that such a metric would provide greater discriminatory power than the contact angle and sought to ascertain whether oil dewetting free energies could resolve differences among surfaces as effectively as dehydration free energies.

Comparing the oil dewetting free energy βΔ*f*_σ_^o^ to the dehydration free energy βΔ*f*_σ_^w^ reveals a weak, positive correlation between oleophobicity and hydrophobicity (Figure 3). This trend is consistent with the expectation that lower surface energy disfavors wetting by both polar and nonpolar liquids. However, βΔ*f*_σ_^o^ exhibits substantially less variation across polymers than βΔ*f*_σ_^w^. The oil dewetting free energies cluster into approximately four data bands: the first includes only amorphous PE, which exhibits the lowest βΔ*f*_σ_^o^ despite its hydrocarbon chemistry; the second includes PTFE-based systems and a frozen amorphous PE surface; the third includes all other polymers except crystalline Nylon-66, which comprises the fourth band. These groupings (highlighted by the background shading in the figure) are not rigorously or precisely defined but merely facilitate observation of apparent trends. The dehydration free energies can also be partitioned into a few bands, but they are more clearly resolved compared to oil dewetting free energies. This reduced sensitivity of βΔ*f*_σ_^o^ ultimately reflects the nonpolar nature of hexadecane, which lacks strong directional interactions such as hydrogen bonding that amplify differences among surfaces in the case of water. Despite the compressed range of oleophobicity values, the observed bands likely reflect distinct physical mechanisms governing oil–surface interactions.

**Figure 3:**
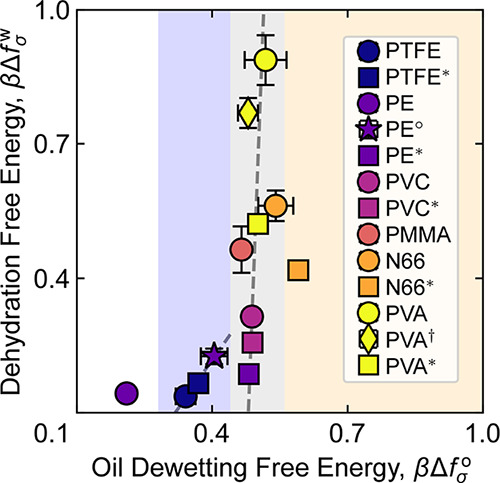
Correlation of oil dewetting and dehydration free energies across different polymer surfaces. The blue, gray, and orange shaded regions highlight four possible data bands. The gray dashed lines are the linear regressions of data points in the blue and gray bands as a guide to the trend. The coefficients of determination for the linear regressions, *R* ^2^, are 0.98 and 0.15. In the legend, * indicates crystal surfaces, ° indicates frozen amorphous surface, ^†^ indicates amorphous isotactic surface. The dehydration free energies of PTFE, PE, PE*, PE°, PVC, PVC*, PMMA, N66, N66*, PVA, PVA^†^ are from ref 82, while dehydration free energies for PTFE* and PVA* are calculated following the same method as ref 82. There is no available data on dehydration free energy for pDVOCB-based systems due to a lack of compatible water model. The error bars reflect statistical uncertainties reported as the standard error of the mean from three independent simulations.

As the sole occupant in the first band, amorphous PE (purple circle) exhibits apparent anomalously low βΔ*f*_σ_^o^. We attribute this result to mechanical deformation rather than surface chemistry. Namely, the soft PE surface deforms to maintain persistent contact with hexadecane during biased simulations, artificially lowering the apparent dewetting free energy. To demonstrate this, we provide a counterfactual in the form of frozen amorphous PE (purple star), which yields βΔ*f*_σ_^o^ more consistent with other polymers in the other bands. A better computation of βΔ*f*_σ_^o^ for soft surfaces may be enabled by reformulating the collective variable. We note that pure amorphous PE is anyhow rarely encountered in practice, as PE possesses a low glass-transition temperature and readily adopts semicrystalline states.

At the opposite extreme band, crystalline Nylon-66 (orange square) exhibits elevated βΔ*f*_σ_^o^. This is due to the formation of highly ordered interfacial hexadecane structures that stabilize the oil layer and increase the energetic penalty for dewetting (Supporting Information, Figure S9), thus making it distinct from amorphous Nylon-66 (orange circle). Between these extremes, the majority of polymer surfaces cluster within a narrow range of βΔ*f*_σ_^o^ despite spanning a wide range of βΔ*f*_σ_^w^; this behavior directly illustrates how dispersion-dominated interactions compress oleophobicity variation relative to hydrophobicity. In the second blue band, comprising PTFE-based systems and frozen PE, there appear to be consistent trends between oil dewetting and hydrophobic character; these are also among the most well-behaved surfaces in the simulations, which display nonzero contact angles with hexadecane. In the third gray band, the direct correlation between hydrophobicity and oleophobicity breaks down because n-hexadecane, as a nonpolar molecule lacking hydrogen-bonding capability, is less sensitive to surface chemistry than water. Collectively, these mechanistic explanations confirm that the narrower range of βΔ*f*_σ_^o^ compared to βΔ*f*_σ_^w^ reflects the physical reality that oleophobicity inherently varies less across polymer chemistries than hydrophobicity, rather than a limitation of the metric.

### 3.3 Efficiency and Limits of a Ghost Probe Energy Metric

To further probe the molecular interactions governing oleophobicity, we introduce the ghost probe energy β*E*_gp_, which quantifies the Lennard-Jones interaction strength between the polymer surface and a probe molecule representing a CH_2_ group of hexadecane. This metric is computed by placing a grid of ghost particles above the surface that interact only with the polymer, not with each other or with bulk hexadecane, and sampling the Boltzmann-weighted interaction energy above the surface (see Section 2.4.3). By isolating the direct surface–oil interaction from oil–oil contributions, we suggest the ghost probe energy as a computationally efficient measure of intrinsic surface affinity for nonpolar molecules.

Overall, the ghost probe energy exhibits a strong linear correlation with the oil dewetting free energy βΔ*f*_σ_^o^ for amorphous surfaces (Figure 4A). This correlation is particularly strong when excluding PE, the behavior of which was rationalized earlier as due to surface deformation, while frozen amorphous PE falls along the linear trend. This strong correlation supports the insight that van der Waals dispersion interactions are the dominant factor governing oleophobicity on flat polymer surfaces. Notably, the ghost probe energy does not require explicit hexadecane molecules in the simulation, reducing the computational cost by approximately 20-fold relative to the calculation of βΔ*f*_σ_^o^ on our resources (2.9 GHz Intel Cascade Lake CPUs). The ghost probe energy thus provides an efficient and practical metric for screening oleophobicity of flat amorphous polymer surfaces.

**Figure 4:**
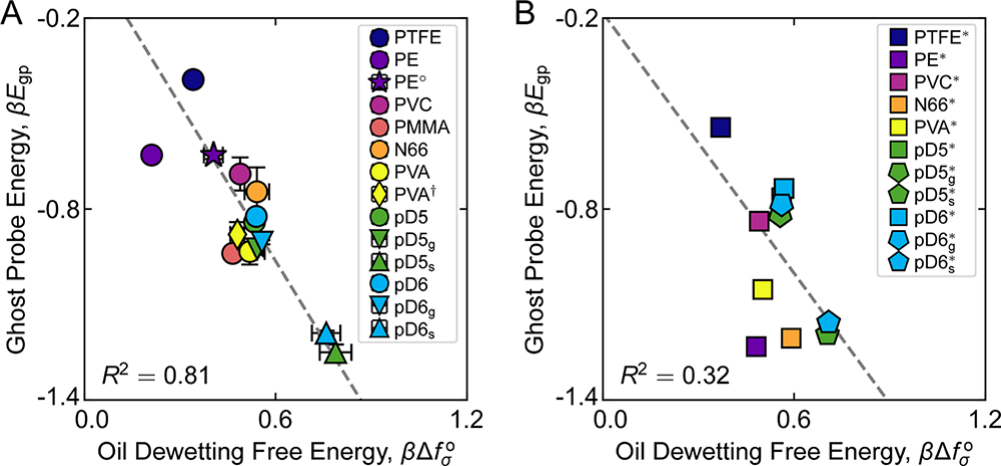
Correlation of oil dewetting free energy and vdW interaction metric of (A) amorphous and (B) crystalline polymer surfaces. The gray dashed lines are the linear regression of data points (excluding amorphous PE surface) as a guide to the trend. Inset numbers correspond to coefficients of determination, *R* ^2^. The error bars reflect statistical uncertainties reported as the standard error of the mean from three independent simulations.

However, the ghost probe energy is seemingly less effective at capturing the physics of the more complex dewetting free energy for many crystalline systems (Figure 4B). By inspection, crystalline PE, PVA, and Nylon-66 notably exhibit lower β*E*_gp_ than expected from their oil dewetting free energies. Figure S9 illustrates the likely origin for these deviations as resulting from the ordered nature of crystalline surfaces. In these cases, the surface chemistry induces hexadecane molecules to adopt crystal-like arrangements at the interface, and this induced ordering results in sustained structural correlations extending approximately 10–20 Å above the polymer surface (corresponding to three to four molecular layers). The lower β*E*_gp_ values for PE, PVA, and Nylon-66 indicate stable adsorption sites that promote ordered hexadecane structures, yet because the ghost probe energy captures only enthalpic interactions, it does not reflect the entropic penalty of displacing this ordered phase. This highlights that additional considerations, beyond simple dispersion forces, are necessary to fully describe oleophobicity on ordered substrates.

### 3.4 Dynamic Characterization of Oleophobicity

The ghost probe energy and oil dewetting free energy characterize the thermodynamics of oil–surface interactions, but the dynamics of interfacial hexadecane also reflect oleophobic character. Surfaces that interact weakly with hexadecane should exhibit both lower dewetting free energies and faster interfacial diffusion, as molecules are less strongly tethered to the surface. This dynamic perspective is particularly relevant to applications requiring rapid oil transport or removal, such as self-cleaning coatings. We thus examine the diffusivity of interfacial hexadecane (Figure 5A) as another means of characterizing oleophobicity.

**Figure 5:**
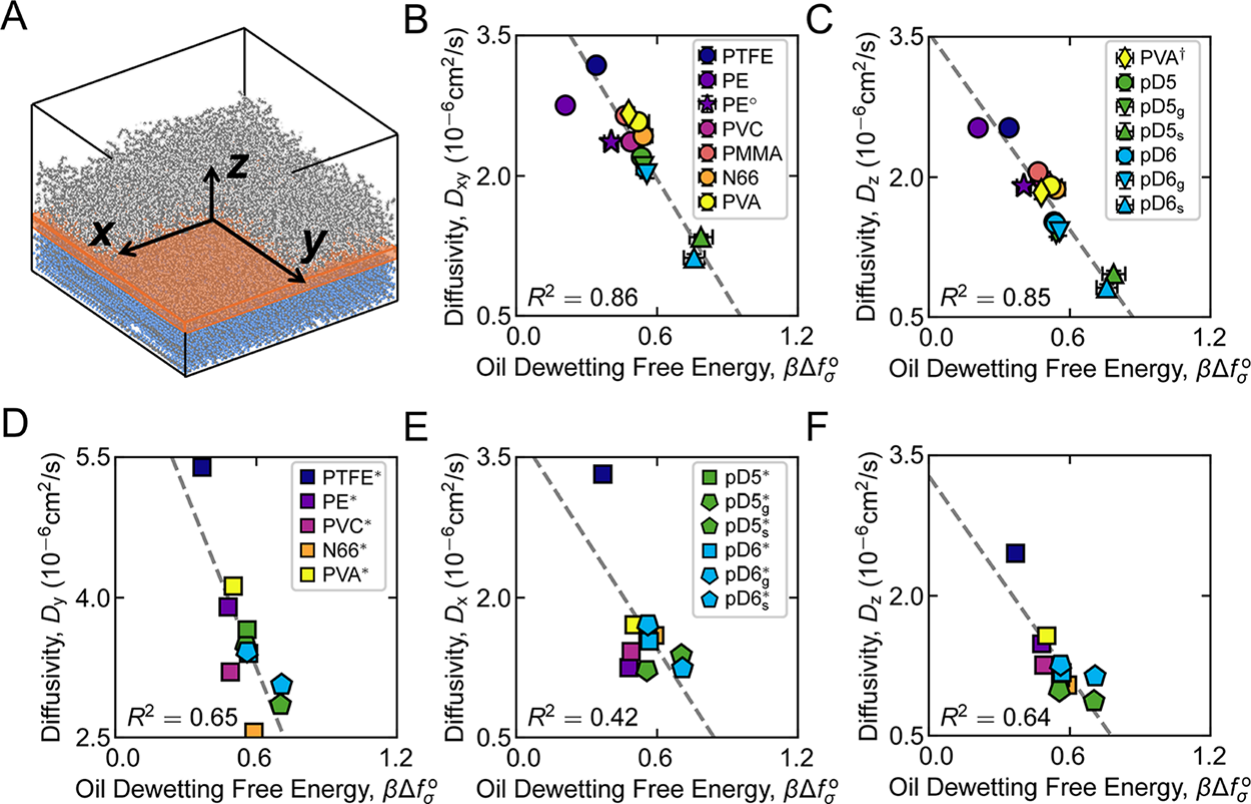
Correlation of thermodynamic and dynamic metrics of oleophobicity. (A) Characteristic system set up. The specific system shown is for PTFE*. Orange shading indicates the region used to calculate interfacial diffusivity. The *x*, *y*, and *z* directions are shown as arrows at the interface. On amorphous surfaces, *x* and *y* directions are equivalent. On crystalline surfaces, polymer chains are aligned in the y direction, except PVC*. (B–F) Comparison of interfacial diffusivity with oil dewetting free energy in specified directions and select surfaces. Panels (B) and (C) correspond to amorphous surfaces, while Panels (D–F) correspond to crystalline surfaces (denoted by ‘*’). The gray dashed lines are the linear regression of data points (excluding amorphous PE surface) as a guide to the trend. Inset numbers correspond to coefficients of determination, *R* ^2^. The error bars reflect statistical uncertainties reported as the standard error of the mean from three independent simulations.

Figure 5B demonstrates that the in-plane diffusivity for amorphous surfaces, *D*_*xy*_, generally decreases with increasing oil dewetting free energy βΔ*f*_σ_^o^. A similar strong correlation is observed for the out-of-plane diffusivity *D*_*z*_, although diffusion along the *z*-direction is slower due to interfacial confinement (Figure 5C). Interestingly, Figures 5D–F shows that crystalline surfaces exhibit qualitatively distinct diffusion behavior. In these cases, the alignment of hexadecane molecules along a preferred direction (e.g., the *y*-direction, Supporting Information, Figure S9) results in pronounced diffusion anisotropy. Diffusion along this preferred direction is not only faster than along the perpendicular direction (e.g., the *x*-direction) but also exceeds the diffusivity on corresponding amorphous surfaces. Most strikingly, on crystalline PTFE, *D*_*y*_ = 5.39 × 10^–6^cm^2^/s, which surpasses even the bulk hexadecane diffusivity of 4.65 × 10^–6^cm^2^/s. This enhanced interfacial mobility, facilitated by ordered surface channels, suggests potential strategies for designing surfaces that enable spontaneous directional oil transport. Despite these differences, diffusivity for crystalline surfaces maintains a correlation with βΔ*f*_σ_^o^ analogous to that observed for amorphous systems, demonstrating the generality of this relationship across surface morphologies.

## 4 Conclusions

In this work, we investigated the oleophobicity (or oleophilicity) of eight polymers, featuring a range of functional chemistry, with hexadecane as a model oil. Using molecular dynamics simulations, four metrics were evaluated: oil contact angle, oil dewetting free energy (βΔ*f*_σ_^o^), a ghost probe energy (β*E*_gp_), and interfacial diffusivity. Oil contact angles proved unreliable for discriminating among surfaces, with experimental values clustering in a narrow range and simulations yielding complete wetting for most polymer surfaces studied due to the low surface tension of hexadecane. The remaining metrics provided more effective characterization and revealed consistent physical trends across the polymer chemistries examined.

We find that oleophobicity exhibits less variation across polymer chemistries than hydrophobicity, which is consistent with established expectations that fabricating superoleophobic surfaces is inherently more challenging than making hydrophobic ones.^4,70,71^ Our results provide molecular-level evidence for this principle and quantitatively connect it to the dominance of nondirectional dispersion interactions in oil wetting. Namely, the strong correlation between the ghost probe energy and oil dewetting free energy confirms that van der Waals interactions are the primary factor governing oleophobicity on flat amorphous surfaces. Crystalline surfaces, however, can induce structural ordering of interfacial oil molecules, leading to deviations from this correlation and pronounced diffusion anisotropy.

These findings indicate that substantially improving oleophobicity through surface chemistry alone is challenging, emphasizing the importance of surface patterning strategies for strong oil repellency. The observed anisotropic diffusion on crystalline surfaces also suggests opportunities for directing oil transport along preferred orientations. Finally, the ghost probe energy provides a computationally efficient metric for polymer oleophobicity and may be useful for screening other oils, substrates, and more complex surface architectures.

## Supplementary Material



## Notes

Discussion of mixing rules; details of the oil contact angle experiments; cylindrical droplet geometry simulations for oil contact angle and ghost probe energy on PE surfaces; full list of experimental oil contact angle results; full table of simulation results; and density distribution of crystalline surfaces (PDF)
