# Supplementary material for: Molecular Dynamics Investigation of Oil Wetting on Synthetic Polymer Substrates
Source: Macromolecules. 2026 Jul 10;59(14):8465–75. doi: 10.1021/acs.macromol.6c00947 (PMC13421959; doi:10.1021/acs.macromol.6c00947)
Supplement: Supplementary file 1 [file ma6c00947_si_001.pdf]

Supporting Information

Molecular Dynamics Investigation of Oil Wetting on Synthetic Polymer Substrates

Hang Zhang,† Sahana V. Sundar,‡ Shawn M. Maguire,‡ Rodney D. Priestley,‡,¶ Emily C. Davidson,‡ and Michael A. Webb*,‡

†*Department of Chemistry, Princeton University, Princeton, New Jersey 08540, United States*

‡*Department of Chemical and Biological Engineering, Princeton University, Princeton, New Jersey 08540, United States*

¶*Princeton Materials Institute, Princeton University, Princeton, New Jersey 08540, United States*

E-mail: mawebb@princeton.edu

Contents

S1 Discussion of Mixing Rules **SI-2**

S2 Details of the Oil Contact Angle Experiments **SI-4**

S3 Cylindrical Droplet Geometry Simulations for Oil Contact Angle **SI-8**

S4 Ghost Probe Energy on PE Surfaces **SI-10**

S5 Experimental Oil Contact Angle Results **SI-11**

S6 Summary of Simulation Results **SI-11**

S7 Density Distribution of Crystalline Surfaces **SI-13**

References **SI-14**

S1 Discussion of Mixing Rules

To simulate the pDVOCB surface with hexadecane molecules, we applied three different mixing rules to generate mixed LJ parameters: the Waldman-Hagler rule, the geometric rule, and a scaled Waldman-Hagler rule.

The Waldman-Hagler rule is the default mixing rule of the TAFFI force field.[1]

$$\epsilon_{ij} = \frac{2\sqrt{\epsilon_i \epsilon_j}\sigma_i^3 \sigma_j^3}{\sigma_i^6 + \sigma_j^6} \tag{S1}$$

$$\sigma_{ij} = \left(\frac{1}{2}(\sigma_i^6 + \sigma_j^6)\right)^{\frac{1}{6}} \tag{S2}$$

The geometric rule is the default mixing rule of the OPLS force field family.[2–4]

$$\epsilon_{ij} = \sqrt{\epsilon_i \epsilon_j} \tag{S3}$$

$$\sigma_{ij} = \sqrt{\sigma_i \sigma_j} \tag{S4}$$

These two mixing rules yield very similar results, which, considering the chemistry of pDVOCB, align with the results from other polymer surfaces.

In addition to these two rules, we applied a scaled Waldman-Hagler rule based on DFT results.

$$\epsilon_{ij} = a_{ij} \frac{2\sqrt{\epsilon_i \epsilon_j}\sigma_i^3 \sigma_j^3}{\sigma_i^6 + \sigma_j^6} \tag{S5}$$

$$\sigma_{ij} = \left(\frac{1}{2}(\sigma_i^6 + \sigma_j^6)\right)^{\frac{1}{6}} \tag{S6}$$

where a_{ij} is a scale factor. Because the LJ parameters of the TAFFI force field are fitted from DFT calculations, we sought to test whether similar DFT calculations could improve LJ parameter mixing. The mixed LJ parameters are first generated by the Waldman-Hagler rule and then multiplied by scale factors based on element combinations, obtained by fitting

the pair energy between DVOCB(3) and hexadecane to DFT results at the ωB97X-D3/def2-TZVP level.[5–8] A total of 864 dimer configurations from MD simulations were used for the fitting, as shown in Figure SS1. The fitted scale factors are

$$a_{ij} = \begin{cases} 1.56575427, & i = \mathrm{C} \in \mathrm{TAFFI}, j = \mathrm{C} \in \mathrm{LOPLS\text{-}AA} \\ 0.63247842, & i = \mathrm{C} \in \mathrm{TAFFI}, j = \mathrm{H} \in \mathrm{LOPLS\text{-}AA} \\ 1.15840760, & i = \mathrm{H} \in \mathrm{TAFFI}, j = \mathrm{C} \in \mathrm{LOPLS\text{-}AA} \\ 0.75038809, & i = \mathrm{H} \in \mathrm{TAFFI}, j = \mathrm{H} \in \mathrm{LOPLS\text{-}AA} \end{cases} \tag{S7}$$

However, subsequent simulation results show that this mixing rule substantially overestimates the interaction strength between surface and oil, demonstrating the complexity of mixing bottom-up and top-down force fields.

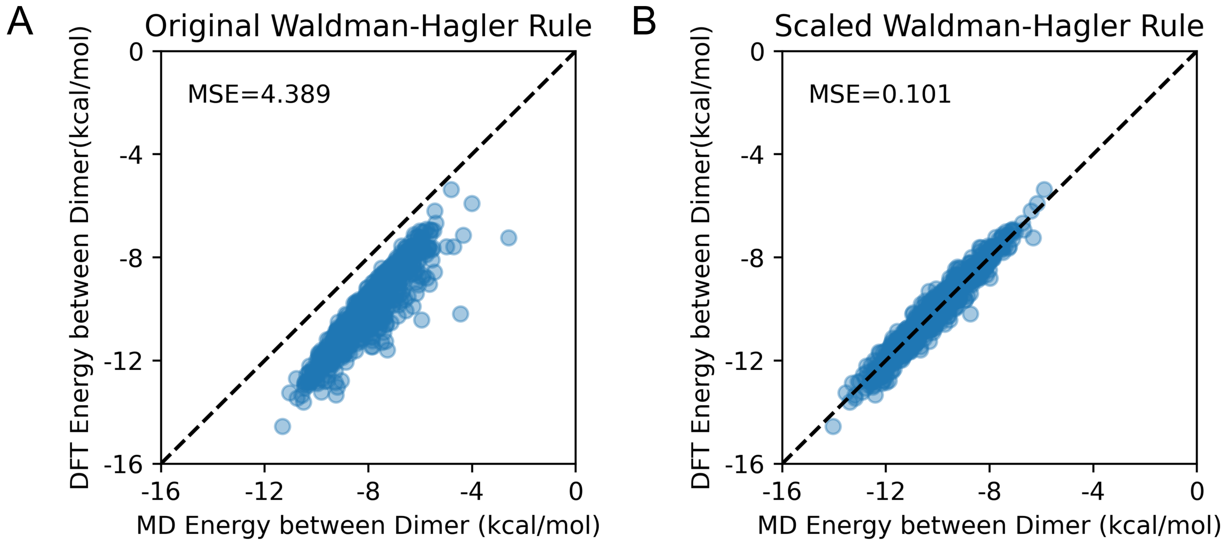

Figure S1: MD vs. DFT energies between DVOCB(3) and hexadecane molecules.

We elected to retain results from all three mixing rules in the main text and treat the results from the geometric rule and scaled Waldman-Hagler rule as arising from model surfaces.

S2 Details of the Oil Contact Angle Experiments

Samples of high-density polyethylene (HDPE; M_w = 66 kg/mol, Đ= 4.2) and low-density polyethylene (LDPE; medium molecular weight, melt index MI = 1.8 dg/min) were provided by Dow, and isotactic polypropylene (iPP; M_w = 550 kg/mol, Đ= 5.0) was provided by Braskem. Samples of poly(vinyl chloride) (PVC) and polytetrafluoroethylene (PTFE) were purchased as commercial sheets from McMaster-Carr. Samples of nylon 6,6 (N66; $M_w \sim 226$ kg/mol), poly(vinyl alcohol) (PVA; $M_w \sim 89$ kg/mol), poly(vinylidene fluoride) (PVDF; $M_w \sim 180$ kg/mol, $M_n \sim 71$ kg/mol), and poly(methyl methacrylate) (PMMA; $M_w \sim 350$ kg/mol) were purchased from Sigma-Aldrich. All homopolymers were used as received from the manufacturers. Hexadecane was purchased commercially and used as received without purification. PVDF and iPP are not studied in the simulations, but contact angle results are listed in Section S5 for comparison.

Polymer sheets were prepared by loading polymer pellets or powders between two Teflon sheets in a Carver melt-press at approximately 10 ℃ above the melting temperature (or glass transition temperature for purely amorphous samples) while applying slight pressure. The pDVOCB(6, 46) samples were prepared by loading powder into a metal shim between two sheets of Kapton to minimize oxidation during melt-pressing. Approximately 2.5 cm × 2.5 cm sections were cut from the resulting polymer sheets and mounted on glass slides for atomic force microscopy (AFM) and contact angle measurements. PVC and PTFE samples were cut from as-purchased commercial sheets instead of melt-pressing. The topography of each sample was measured with an Asylum Research MFP-3D Stand Alone (MFP-3D-SA) AFM using non-contact tips (Bruker RTESP-300, tip radius of approximately 10 nm). For each sample, three 20 µm × 20 µm scans were taken at different locations on the surface to collect data for average roughness calculations. All AFM images were processed using Gwyddion software to extract root-mean-square (RMS) roughness values and rugosity factors.

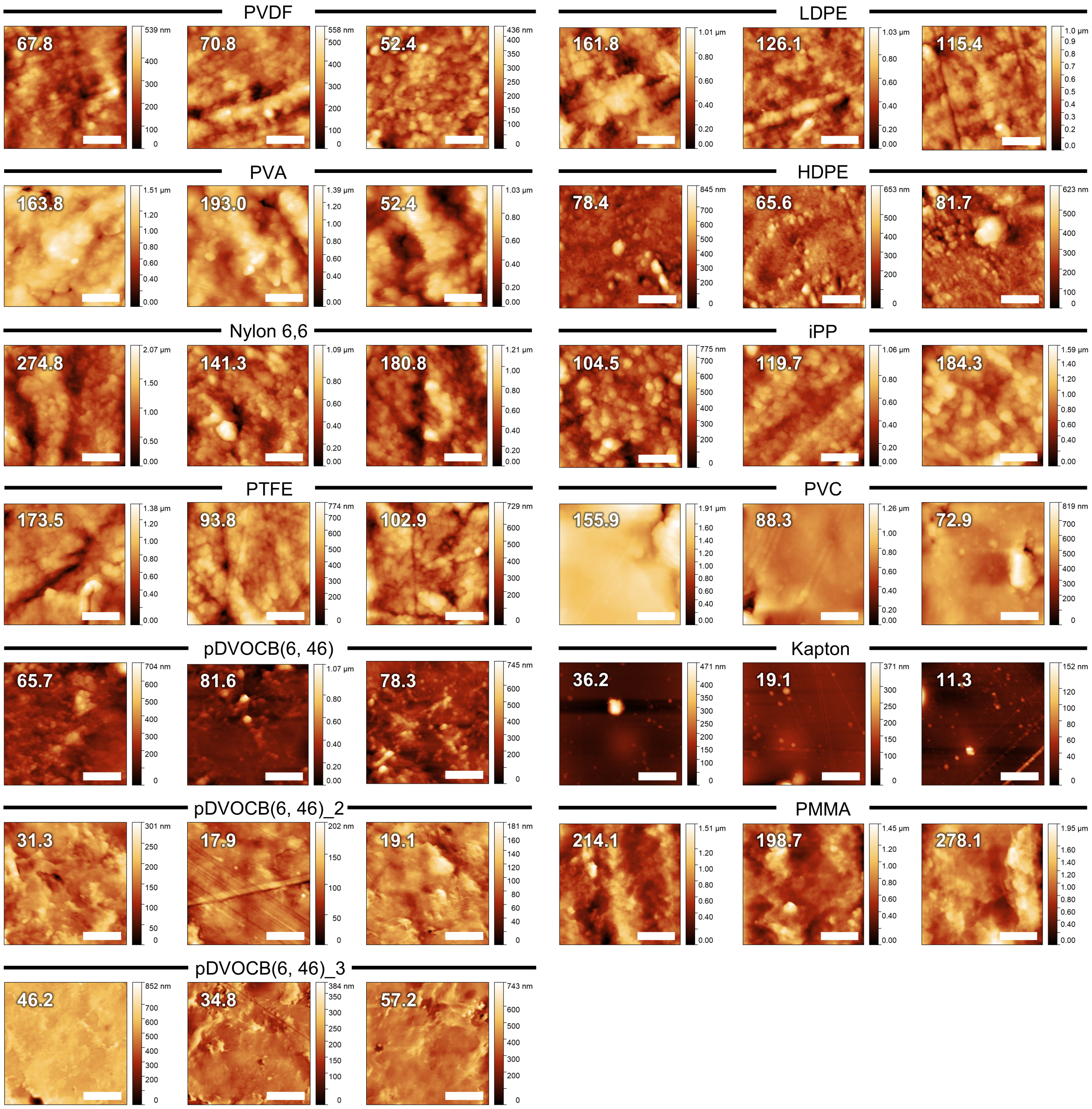

Figure S2: Atomic force microscopy (AFM) measurements taken on polymer surfaces used for contact angle measurements. For each prepared surface, three measurements were taken and used to calculate average surface roughness using Gwyddion software. All scale bars are 5 microns. The RMS roughness from each scan (in nm) is noted in the top left corner.

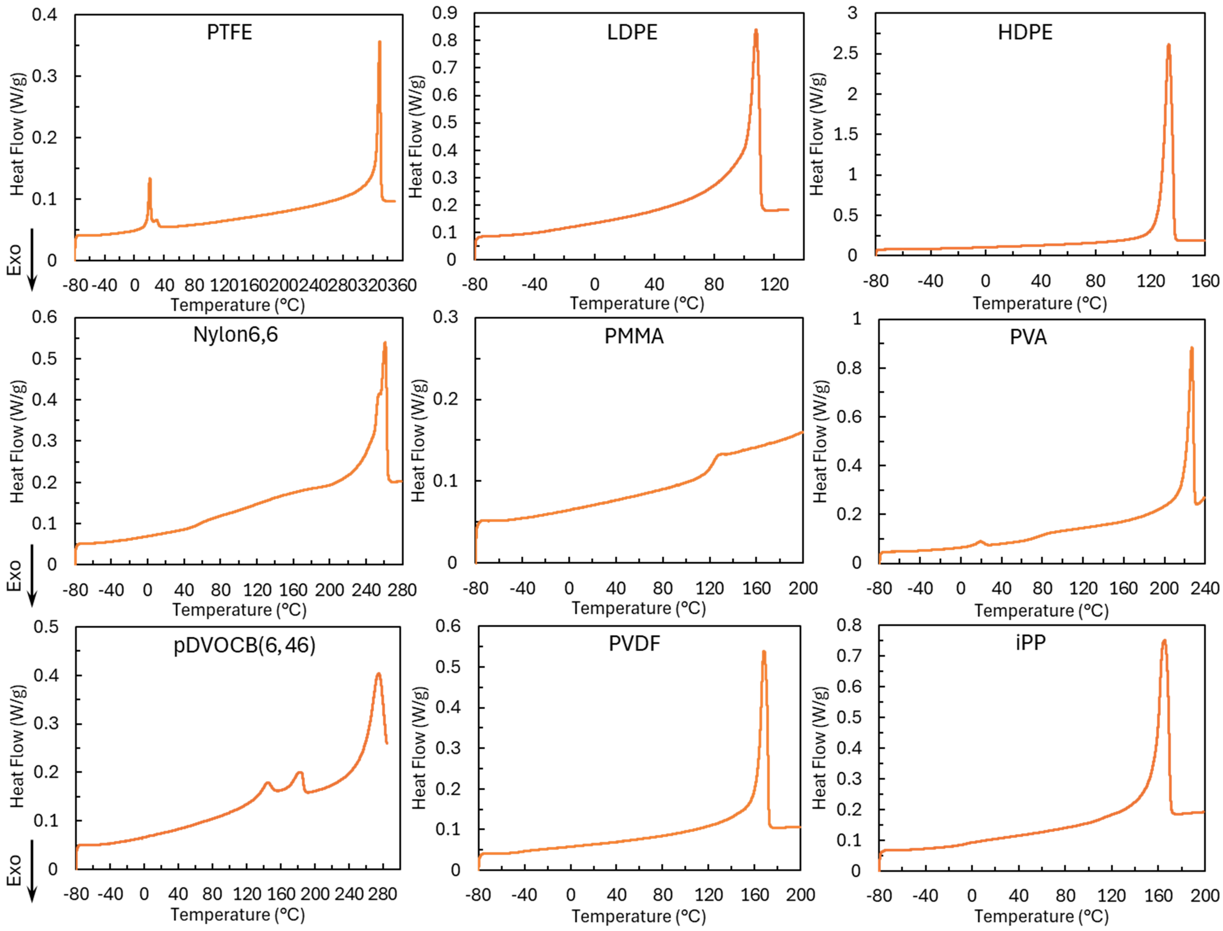

Figure S3: Differential scanning calorimetry (DSC) data for polymers used for contact angle measurements. All data shown is the second heating curve at a ramp rate of 5 °C/min. Note: DSC second heating data for polyvinyl chloride is not included due to significant polymer degradation after the first melting.

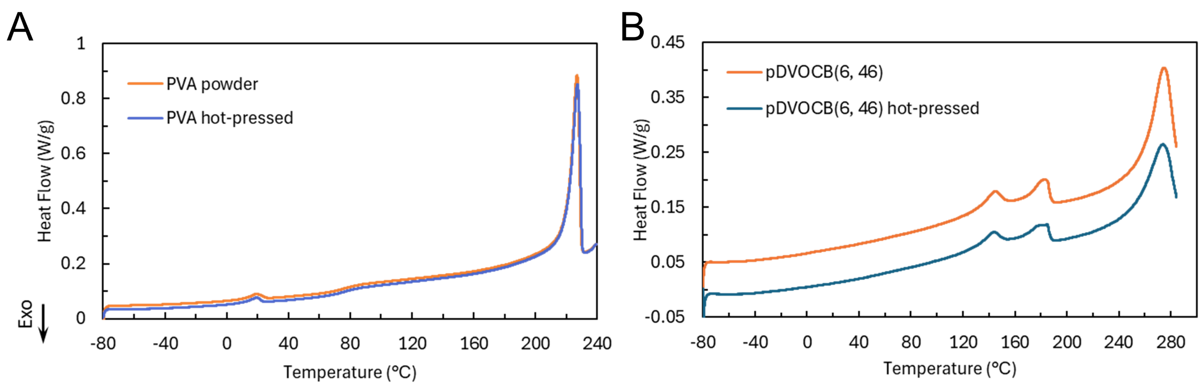

Figure S4: DSC data for (A) PVA and (B) pDVOCB(6, 46) polymer powder (orange curves) vs melt-pressed polymer (blue curves) as prepared for contact angle measurements. DSC data shown is taken from the second heating curve at a ramp rate of 5 °C/min.

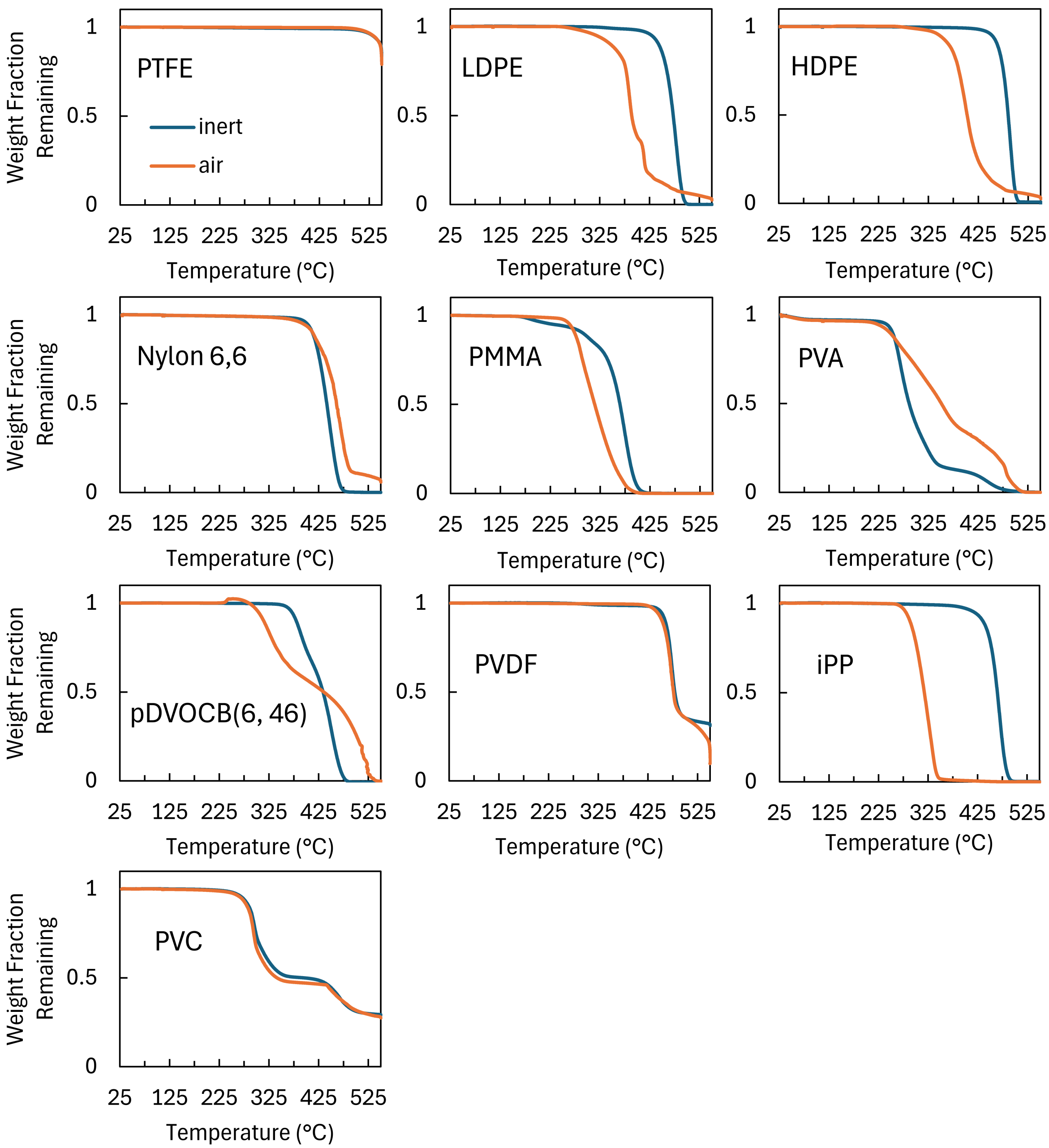

Figure S5: Thermogravimetric analysis (TGA) data for polymers used for contact angle measurements under inert (blue curves) and air (orange curves) environments. All TGA data shown is taken at a heating rate of 20 °C/min.

S3 Cylindrical Droplet Geometry Simulations for Oil Contact Angle

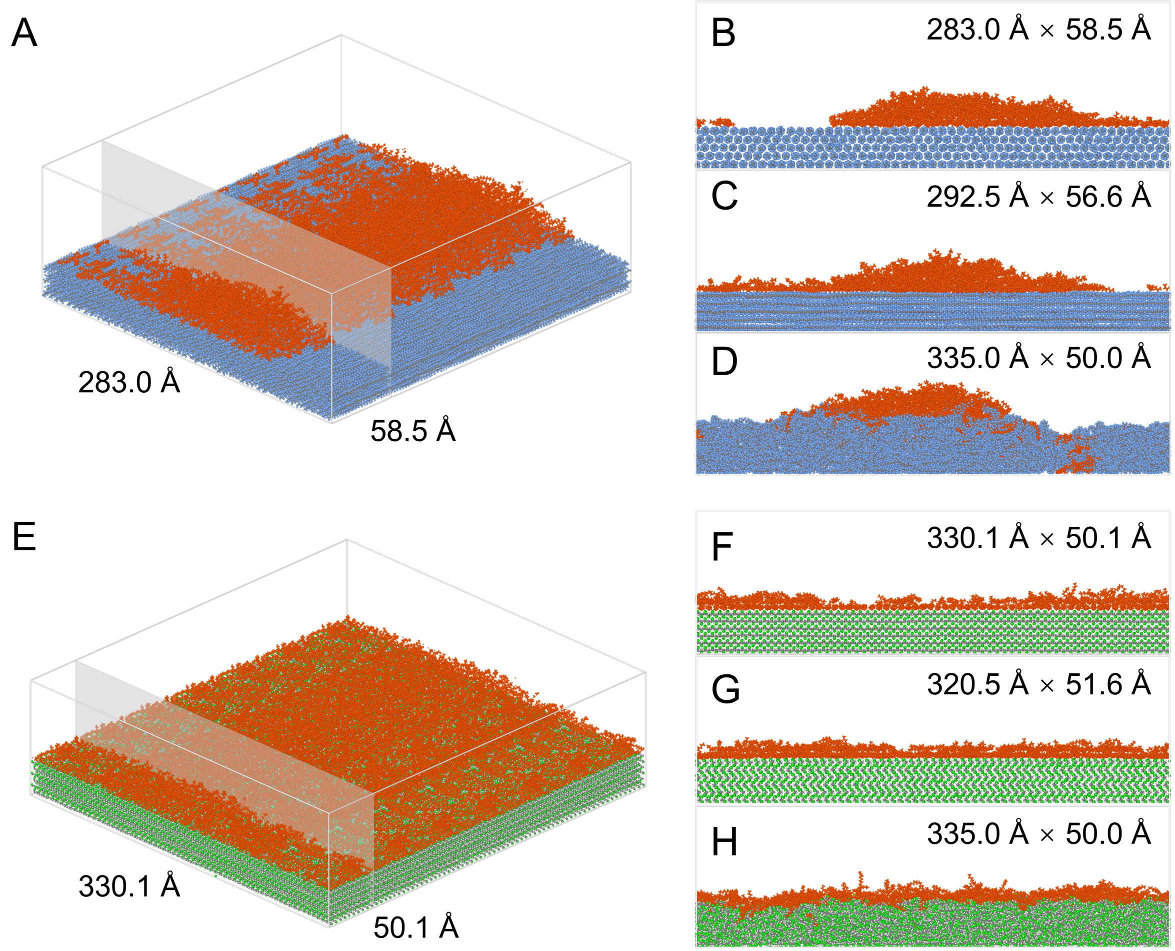

Figure S6: Wetting behavior in cylindrical droplet geometry simulations. (A) Snapshot of the crystalline PTFE simulation box, for which the polymer chain direction is parallel to the cylindrical droplet axis. The simulation box is repeated five times along the cylindrical direction for clarity. A white semi-transparent plane indicates a single simulation box. (B–D) Side-view snapshots of the crystalline PTFE and amorphous PTFE simulation boxes. The calculated contact angles are 23.3°, 24.0°, and 32.8°, respectively. (E) Snapshot of the crystalline PVC simulation box, for which the polymer chain direction is perpendicular to the cylindrical droplet axis. The simulation box is repeated seven times along the cylindrical direction for clarity. A white semi-transparent plane indicates a single simulation box. (F–H) Side-view snapshots of the crystalline PVC and amorphous PVC simulation boxes. The insets show the simulation box dimensions in the xy directions. Molecular images are visualized using OVITO.[9] Polymer atoms are colored as follows: carbon is gray, fluorine is blue, chlorine is green, and hydrogen is white. Oil atoms are colored orange for clarity.

To test whether the line-tension contribution described by the modified Young's equa-

tion significantly affects the oil contact angles in our simulations, we performed additional cylindrical droplet geometry simulations. This geometry has been shown in previous studies to reduce finite-size and line-tension effects in contact-angle calculations.[10,11] The systems were prepared use the same process as other polymer surfaces. 256 oil molecules were used to form the droplet. 40 ns NVT simulations were performed and the last 10 ns simulations were used to calculate contact angle by fitting half density contours to an arc. As shown in Figure S6, the PTFE systems retain nonzero contact angles, whereas the PVC systems exhibit complete wetting in the cylindrical droplet simulations. The calculated contact angles for the PTFE systems are close to those obtained from the spherical droplet geometry simulations, indicating that the wetting behavior observed here is not primarily controlled by line-tension effects.

S4 Ghost Probe Energy on PE Surfaces

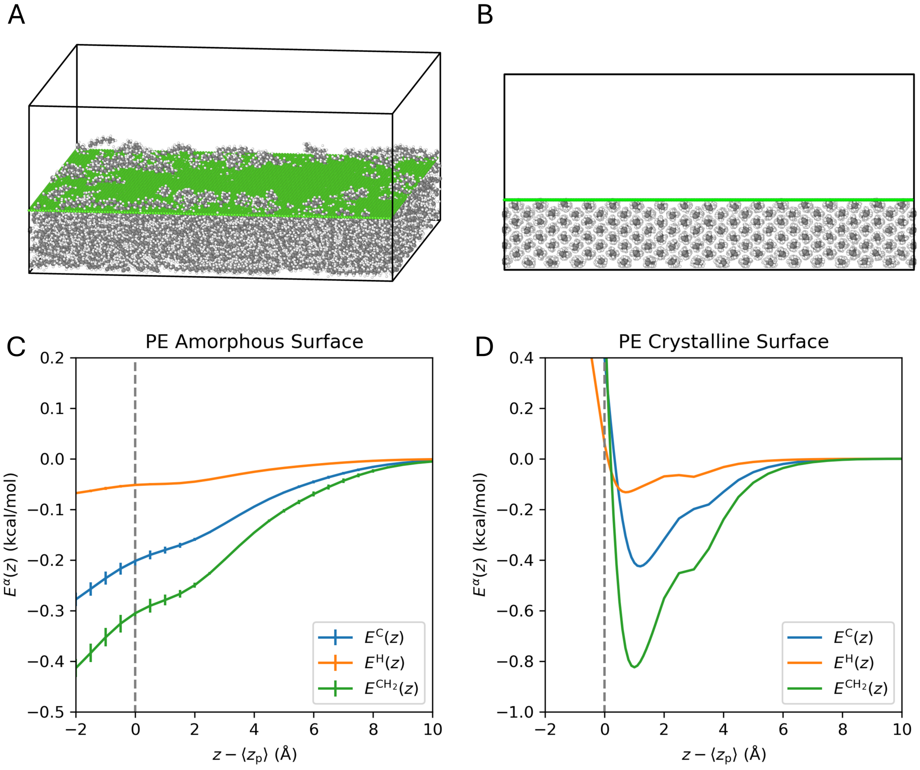

Figure S7: (A) Snapshot of the amorphous PE surface with a grid of ghost particles at $\langle z_p \rangle$. (B) Snapshot of the crystalline PE surface with a grid of ghost particles at the height of the minimum. (C) $E^\alpha(z)$ of amorphous PE as a function of ghost particle height z. (D) $E^\alpha(z)$ of crystalline PE as a function of ghost particle height z. The gray dashed lines indicate the interface position defined by $\langle z_p \rangle$. The error bars reflect statistical uncertainties reported as the standard deviation from three independent sample simulations.

The variation of $E^\alpha(z)$ with height above the surface depends on the surface geometry. For amorphous surfaces, $E^\alpha(z)$ gradually decreases as the grid of ghost particles approaches the surface (Figure S7A). For crystalline surfaces, $E^\alpha(z)$ exhibits a minimum at z close to the interface (Figure S7B). Additional simulations with 0.1 Å step size were performed near the minimum region to improve the quality of spline interpolation.

S5 Experimental Oil Contact Angle Results

Experimental oil contact angles are relatively small across all surfaces, as shown in Figure S8. PVDF and iPP were not studied in the simulations. The contact angle of LDPE is shown for PE in the main text because it is more similar to amorphous PE in the simulations. pDVOCB(6,46) exhibited relatively low contact angles in the experiments comparing to other polymers, which could arise from oxidation or cross-linking of pDVOCB(6,46) during melt-processing. PVA raises similar concerns. The DSC data for these two polymers in powder and hot-pressed samples are shown in Figure S4. The PVC sample was purchased as a commercial sheet, which often has large amount of plasticizers and additives that could influence the oil contact angle. Further investigation would be needed to address these concerns regarding the samples.

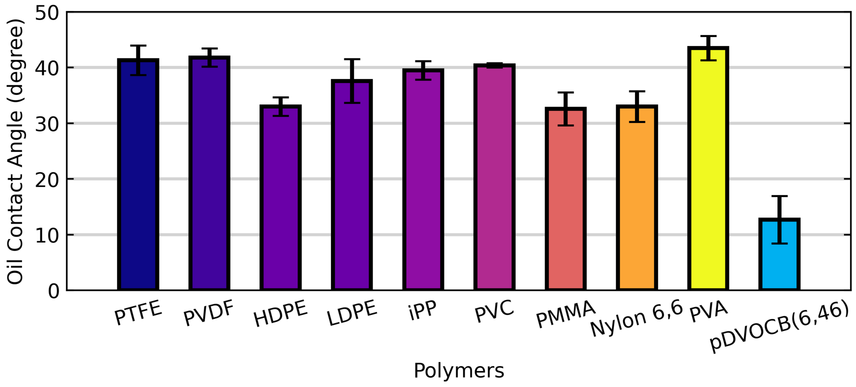

Figure S8: Experimental oil contact angle results. The error bars reflect statistical uncertainties reported as the standard deviation of replicate contact angle measurements.

S6 Summary of Simulation Results

For ease of reference, all simulation results from this work are shown in Table S1.

Table S1: Summary of Simulation Results

Polymer Surface	Oil Contact Angle (°)	Dehydration Free Energy[a]	Oil Dewetting Free Energy	Ghost Probe Energy	Oil Diffusion Coefficient in x, y, and z directions ($10^{-7}cm^2/s$)[b]		
PTFE Amor.	33.0±1.3	0.137±0.012	0.341±0.022	-0.1.64±0.004	31.85±0.27	31.85±0.27	25.30±0.26
PTFE Crys.	21.7	0.166	0.369	-0.214	33.19	53.93	24.57
PE Amor.	0	0.143±0.013	0.210±0.007	-0.244±0.004	27.55±0.58	27.55±0.58	25.29±0.54
PE Amor. Frozen	17.9±0.7	0.226±0.018	0.404±0.030	-0.244±0.004[c]	23.63±0.40	23.63±0.40	19.05±0.42
PE Crys.	0	0.187	0.481	-0.444	12.49	39.02	14.87
PVC Atactic Amor.	0	0.314±0.007	0.488±0.005	-0.263±0.017	23.65±0.42	23.65±0.42	19.69±0.91
PVC Syndiotactic Crys.	0	0.257	0.490	-0.313	14.20	32.03	12.55
PMMA Atactic Amor.	0	0.464±0.052	0.465±0.005	-0.346±0.007	26.47±0.30	26.47±0.30	20.60±0.56
Nylon 6,6 Amor.	0	0.562±0.034	0.541±0.038	-0.282±0.026	24.28±0.82	24.28±0.82	18.76±0.38
Nylon 6,6 Crys.	0	0.418	0.591	-0.435	15.94	25.59	10.42
PVA Atactic Amor.	0	0.888±0.056	0.518±0.047	-0.345±0.014	25.80±0.18	25.80±0.18	19.09±0.05
PVA Isotactic Amor.	0	0.769±0.034	0.479±0.022	-0.327±0.013	26.66±0.17	26.66±0.17	18.37±0.73
PVA Atactic Crys.	0	0.521	0.501	-0.384	17.11	41.23	15.71
pDVOCB(5,40) Amor. (WH)	0	—	0.533±0.012	-0.314±0.005	22.03±0.08	22.03±0.08	15.26±0.26
pDVOCB(5,m) Crys. (WH)	0	—	0.561	-0.288	16.27	36.56	11.22
pDVOCB(6,46) Amor. (WH)	0	—	0.539±0.009	-0.308±0.002	20.58±0.39	20.58±0.39	15.07±0.18
pDVOCB(6,m) Crys. (WH)	0	—	0.567	-0.278	15.31	33.98	11.75
pDVOCB(5,40) Amor. (Geo)	0	—	0.542±0.019	-0.342±0.006	21.02±0.20	21.02±0.20	13.99±0.33
pDVOCB(5,m) Crys. (Geo)	0	—	0.556	-0.306	12.21	34.85	9.84
pDVOCB(6,46) Amor. (Geo)	0	—	0.557±0.007	-0.335±0.002	20.24±0.47	20.24±0.47	14.18±0.43
pDVOCB(6,m) Crys. (Geo)	0	—	0.560	-0.295	17.15	34.2	12.61
pDVOCB(5,40) Amor. (SWH)	0	—	0.787±0.049	-0.450±0.008	13.50±0.28	13.50±0.28	9.67±0.35
pDVOCB(5,m) Crys. (SWH)	0	—	0.704	-0.431	13.82	28.51	8.69
pDVOCB(6,46) Amor. (SWH)	0	—	0.758±0.044	-0.430±0.003	11.27±0.39	11.27±0.39	8.14±0.35
pDVOCB(6,m) Crys. (SWH)	0	—	0.708	-0.419	12.42	30.67	11.37

The uncertainties are reported as the standard error of the mean from three independent sample simulations.

[a] All results except "PTFE Crystalline Surface" and "PVA Atactic Crystalline Surface" are from Ref 12.

[b] For amorphous surface, the diffusion coefficients in x- and y-directions are averaged.

[c] The Ghost Probe Energy of PE is used for PE°.

— Data not available.

S7 Density Distribution of Crystalline Surfaces

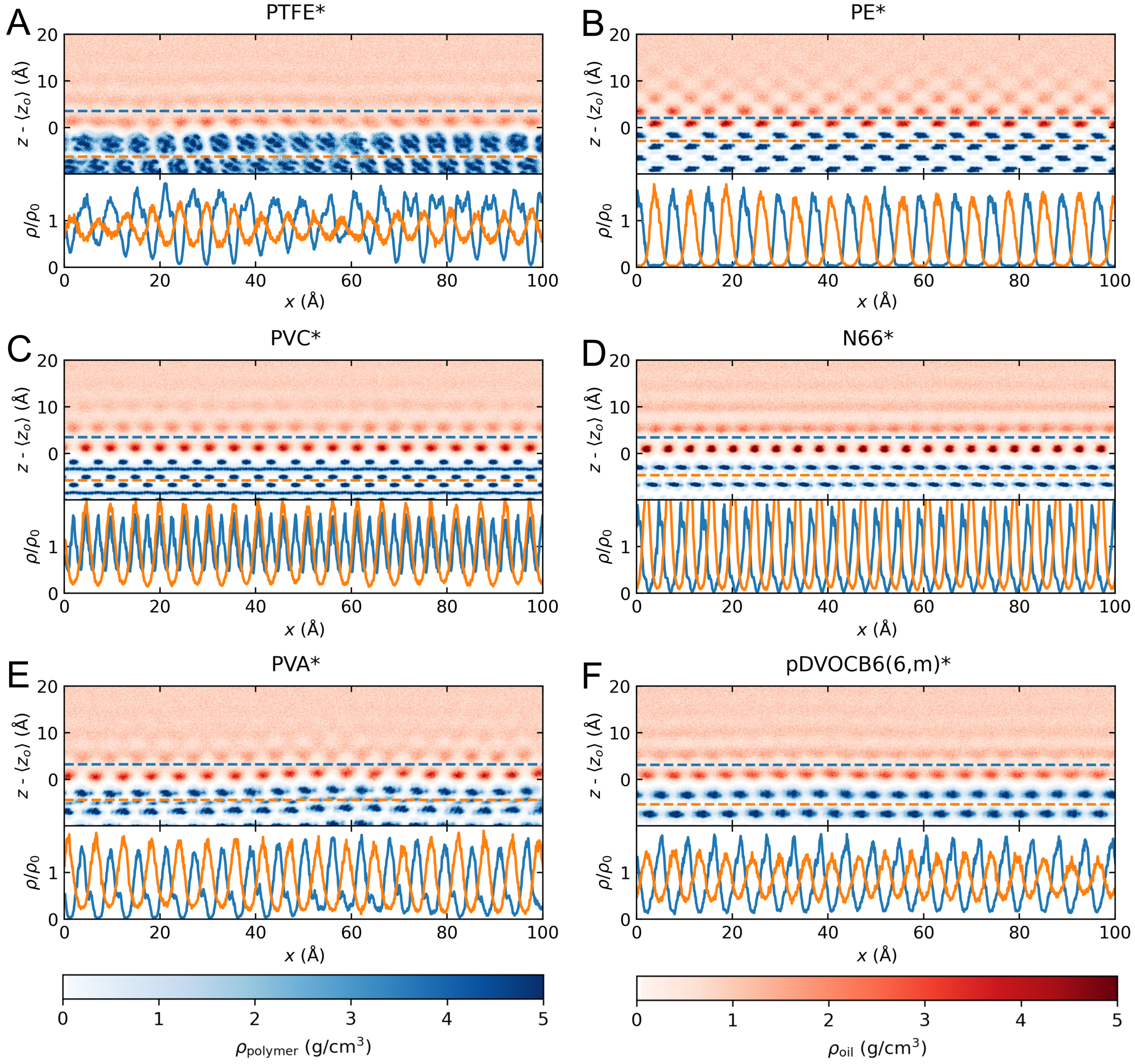

Figure S9: Polymer and oil density distributions in the xz plane and along the x axis near the interface for (A) PTFE*, (B) PE*, (C) PVC*, (D) N66*, (E) PVA*, and (F) pD6*. All pDVOCB systems yield similar results; therefore, only pD6* is shown. In the upper portion of each panel, the blue and orange dashed lines indicate the region used to calculate the density distribution along the x axis shown in the lower portion.

References

(1) Seo, B.; Lin, Z.-Y.; Zhao, Q.; Webb, M. A.; Savoie, B. M. Topology Automated Force-Field Interactions (TAFFI): A Framework for Developing Transferable Force Fields. *Journal of Chemical Information and Modeling* **2021**, *61*, 5013–5027, DOI: 10.1021/acs.jcim.1c00491.

(2) Jorgensen, W. L.; Maxwell, D. S.; Tirado-Rives, J. Development and Testing of the OPLS All-Atom Force Field on Conformational Energetics and Properties of Organic Liquids. *Journal of the American Chemical Society* **1996**, *118*, 11225–11236, DOI: 10.1021/ja9621760.

(3) Siu, S. W.; Pluhackova, K.; Böckmann, R. A. Optimization of the OPLS-AA force field for long hydrocarbons. *Journal of Chemical Theory and Computation* **2012**, *8*, 1459–1470, DOI: 10.1021/ct200908r.

(4) Pluhackova, K.; Morhenn, H.; Lautner, L.; Lohstroh, W.; Nemkovski, K. S.; Unruh, T.; Böckmann, R. A. Extension of the LOPLS-AA Force Field for Alcohols, Esters, and Monoolein Bilayers and its Validation by Neutron Scattering Experiments. *Journal of Physical Chemistry B* **2015**, *119*, 15287–15299, DOI: 10.1021/acs.jpcb.5b08569.

(5) Lin, Y.-S.; Li, G.-D.; Mao, S.-P.; Chai, J.-D. Long-Range Corrected Hybrid Density Functionals with Improved Dispersion Corrections. *Journal of Chemical Theory and Computation* **2012**, *9*, 263–272, DOI: 10.1021/ct300715s.

(6) Grimme, S.; Antony, J.; Ehrlich, S.; Krieg, H. A consistent and accurateab initioparametrization of density functional dispersion correction (DFT-D) for the 94 elements H-Pu. *The Journal of Chemical Physics* **2010**, *132*, DOI: 10.1063/1.3382344.

(7) Schäfer, A.; Horn, H.; Ahlrichs, R. Fully optimized contracted Gaussian basis sets for atoms Li to Kr. *The Journal of Chemical Physics* **1992**, *97*, 2571–2577, DOI: 10.1063/1.463096.

(8) Weigend, F.; Ahlrichs, R. Balanced basis sets of split valence, triple zeta valence and quadruple zeta valence quality for H to Rn: Design and assessment of accuracy. *Physical Chemistry Chemical Physics* **2005**, *7*, 3297, DOI: 10.1039/b508541a.

(9) Stukowski, A. Visualization and analysis of atomistic simulation data with OVITO–the Open Visualization Tool. *Modelling and Simulation in Materials Science and Engineering* **2009**, *18*, 015012, DOI: 10.1088/0965-0393/18/1/015012.

(10) Weijs, J. H.; Marchand, A.; Andreotti, B.; Lohse, D.; Snoeijer, J. H. Origin of line tension for a Lennard-Jones nanodroplet. *Physics of Fluids* **2011**, *23*, DOI: 10.1063/1.3546008.

(11) Kanduč, M. Going beyond the standard line tension: Size-dependent contact angles of water nanodroplets. *Journal of Chemical Physics* **2017**, *147*, 174701, DOI: 10.1063/1.4990741.

(12) Zhang, H.; Sundaresan, S.; Webb, M. A. Molecular Dynamics Investigation of Nanoscale Hydrophobicity of Polymer Surfaces: What Makes Water Wet? *The Journal of Physical Chemistry B* **2023**, *127*, 5115–5127, DOI: 10.1021/acs.jpcb.3c00616.
